# Students’ perceptions of school sugar-free, food and exercise environments enhance healthy eating and physical activity

**DOI:** 10.1017/S1368980021004961

**Published:** 2022-07

**Authors:** Chieh-Hsing Liu, Fong-Ching Chang, Yu-Zhen Niu, Li-Ling Liao, Yen-Jung Chang, Yung Liao, Shu-Fang Shih

**Affiliations:** 1 Department of Health Promotion and Health Education, National Taiwan Normal University, 162, Ho-Ping E. Rd., Sec.1, Taipei 10610, Taiwan; 2 Office of Student Affairs, National Yang Ming Chiao Tung University, Hsinchu, Taiwan; 3 Department of Health Management, I-Shou University, Kaohsiung City, Taiwan; 4 Department of Health Administration, Virginia Commonwealth University, Richmond, USA

**Keywords:** School, Environment, Healthy eating, Physical activity

## Abstract

**Objective:**

The objective of this study was to examine the relationships between students’ perceptions of their school policies and environments (i.e. sugar-sweetened beverages (SSB) free policy, plain water drinking, vegetables and fruit eating campaign, outdoor physical activity initiative, and the SH150 programme (exercise 150 min/week at school)) and their dietary behaviours and physical activity.

**Design:**

Cross-sectional study.

**Setting:**

Primary, middle and high schools in Taiwan.

**Participants:**

A nationally representative sample of 2433 primary school (5th–6th grade) students, 3212 middle school students and 2829 high school students completed the online survey in 2018.

**Results:**

Multivariate analysis results showed that after controlling for school level, gender and age, the students’ perceptions of school sugar-free policies were negatively associated with the consumption of SSB and positively associated with consumption of plain water. Schools’ campaigns promoting the eating of vegetables and fruit were positively associated with students’ consumption of vegetables. In addition, schools’ initiatives promoting outdoor physical activity and the SH150 programme were positively associated with students’ engagement in outdoor physical activities and daily moderate-to-vigorous physical activity.

**Conclusions:**

Students’ perceptions of healthy school policies and environments promote healthy eating and an increase in physical activity for students.

Childhood obesity is considered an epidemic and is an emerging public health problem in many countries. Unhealthy dietary behaviours (i.e. the consumption of sugar-sweetened beverages (SSB) and inadequate consumption of fruits and vegetables) and physical inactivity are both known to have a significant impact on the likelihood of overweight/obesity among children and adolescents^(1)^. Studies have associated regular consumption of SSB with a greater risk of obesity, type 2 diabetes, CHD^(2)^, fatty liver diseases^(3)^ and attention-deficit hyperactivity disorder^(4)^, while a physically inactive lifestyle from youth to adulthood has been associated with an increased risk of impaired glucose metabolism in adulthood^(5)^. The WHO has called for governments to take a leadership role in tackling obesity through remedying obesogenic environments and has suggested that the complexity of obesity mandates a comprehensive approach involving government, schools, parents, civil society and the private sector^(6)^. The prevention of obesity in childhood and adolescence guidelines is augmented by suggestions that combining behaviour-based programmes with environment-based prevention to counteract an ‘obesogenic environment’ is crucial for obesity prevention^(7)^.

The burden of non-communicable diseases (NCD) is high in many countries, and a Global School-based Student Health Survey (2007–2016) that analysed eighty-nine countries showed 82 % of adolescents had more than two lifestyle risk factors for NCD^(8)^. A survey conducted in forty-nine low- and-middle-income countries found that more than 70 % of adolescents had not met the WHO guidelines for either fruit and vegetable consumption or physical activity, while in Pacific island countries and territories half of the students surveyed reported consuming SSB daily^(9)^. In a Canadian study, more than half of the students canvassed did not achieve the recommended level of daily moderate-to-vigorous physical activity^(10)^, while a study conducted in Australia found that 86 % of adolescents did not meet the recommended guidelines for vegetable intake^(11)^. The US National Youth Risk Behaviour Survey found that about half of high school students reported drinking plain water less than three times a day^(12)^.

Many studies support the effectiveness of school-based interventions for preventing childhood obesity^(13)^, and policies that promote a comprehensive school physical activity environment and a healthier school food environment have been associated with a significantly lower risk of obesity^(14)^. A review of studies has shown that school legislative/environmental interventions have had a high success rate (90 %) in reducing the consumption of SSB by adolescents compared with educational/behavioural interventions (65 %)^(15)^, while another review study also found that school food environment policies improved dietary behaviours^(16)^. A Canadian study showed that school nutrition policies had a positive effect on diet quality and healthy beverage consumption^(17)^, while studies in the USA and Mexico have shown that programmes aimed at promoting the drinking of plain water in schools have increased students’ water consumption^(18)^. Another US study indicated that a national school fruits and vegetables provision and SSB restriction policies implemented in elementary, middle and high schools improved the diets and BMI in children, which was associated with decreasing cardiometabolic mortality later in life^(19)^.

Childhood obesity in Taiwan is an emerging public health issue, and the Taiwan Nutrition and Health Survey (2015–2018) estimated the prevalence of overweight and obesity among elementary, middle and high school students at 26·1, 24·7 and 30·2 %, respectively^(20)^. The Taiwan government required all primary and middle schools to implement the Health Promoting Schools approach in 2008^(21)^. Taiwan required elementary and middle schools to restrict the selling of SSB and to implement a school lunch programme. However, students brought SSB to schools, and teachers offered SSB as a reward or an incentive for students’ achievements, and vegetables made up a large portion of the food waste from school lunches. To promote students’ healthy eating, some counties and schools in Taiwan implemented a sugar-free school policy that banned students from bringing SSB to schools and restricted the habit of teachers offering SSB as a reward. Some schools encouraged students to drink plain water rather than SSB and to eat five fruits and vegetables a day. In addition, obesity and myopia are serious problems among children and adolescents in Taiwan. The government initiated the SH150 programme (Sports and Health programme of exercise for at least 150 min at school per week) and an outdoor activity campaign to encourage students to engage in outdoor physical activities during school recess. Some schools implemented ‘85 210 strategies’ (8: 8 h of sleep; 5: 5 or more portions of fruits and vegetables a day; 2: 2 h or less of screen time; 1: 1 h or more of physical activity and 0: 0 sugary drinks, more water) to promote healthy weight among students.

Despite studies examining the effects of healthy school policies on students’ healthy behaviours, few studies have focused on examining the influence of students’ perceptions of school health environments on healthy behaviours. A review study found that school policies influenced students’ healthy behaviours indirectly, and mostly via the school’s social environment, which was perceived by students as creating a wider ‘culture’ of physical activity within the school. That study suggested that future studies should explore the implementation of school policy environments and should address a wider cultural shift in healthy behaviours within schools^(22)^. One study indicated that student awareness of a school fruit and vegetable campaign/programme was associated with an increase in fruit and vegetable consumption^(23)^, while another study also found that students’ perception of the healthfulness of school lunches was positively associated with participation but not with objective school lunch healthfulness^(24)^. Thus, the present study examined the relationships between students’ perceptions of school policies/campaigns (i.e. sugar-free policy, vegetables and fruit eating, and exercise environments) and their healthy behaviours (i.e. dietary behaviour and physical activity).

## Methods

### Participants

Nationally representative samples of primary school students (5th–6th grade), middle school students (7th–9th grade) and high school students (10th–12th grade) were included. A probability-proportionate-to-size sampling method was used to systematically draw a random sample of schools. Three to four classes were randomly selected from each school. We petitioned seventy primary schools, forty-three middle schools and thirty-six high schools to participate in this study, and received confirmation from sixty-two primary schools, forty-one middle schools and thirty-two high schools. Since Taiwan school lunch programmes and school food and beverage sales policies were different at primary, middle and high schools and at different cities/counties, the degree of the implementation of health policies and campaigns in these schools also were different. A total of 2433 primary school students (5th–6th grade), 3212 middle school students (7th–9th grade) and 2829 high school students (10th–12th grade) completed the online survey in 2018. The questionnaire used was online and self-administered.

### Instruments

A self-administered questionnaire was developed based on prior studies such as the Global School-based Student Health Survey^(25)^, Youth Risk Behavior Surveillance System^(26)^, School Health Profiles^(27)^, and Perceptions of the Environment and Patterns of Diet at School Survey^(28)^ to assess students’ perceptions of their school environment and their healthy behaviours. Experts from the fields of public health, health education and nutrition were invited to assess the content validity of the questionnaire. Experts reviewed the draft questionnaire and provided comments and suggestions for improvements. In addition, pretesting surveys were conducted at a primary school (5th–6th grade students), a middle school and a high school to examine students’ responses to the online survey and to evaluate the reliability of the data yielded by the questionnaire. Pretesting results provided feedback on the content and length of the questionnaire. Some questions were revised to increase students’ understanding and correct interpretations and some questions were removed to reduce the burden on students. Approval was obtained from the Institutional Review Board at National Taiwan Normal University.

### Perception of school environments

Student perceptions of their healthy eating and their physical activity environment were measured using six items by adapting questions from prior studies^(27,28)^. Students were asked during the past year whether their school implemented policies, campaigns, programmes or activities for them. Sample statements were as follows: ‘School bans sugar-sweetened beverages in school’; ‘School implements refusing to drink sugar-sweetened beverages campaign’; ‘School implements drinking enough plain water daily campaign’; ‘School implements eating 5 servings of vegetables and fruit a day and eating all vegetables at school lunch campaign’; ‘School implements outdoor physical activity 120 (encourage students taking outdoor physical activity at school recess, 120 min/d)’; and ‘School implements SH150 programme (exercise 150 min/week at school)’. The response options for these items were either ‘yes’ (scoring 1) or ‘no’ (scoring 0).

### Dietary behaviours

Students’ dietary behaviours included eating vegetables, drinking plain water and behaviours regarding the drinking of SSB were measured by adapting questions from prior studies^(26,29)^. Students were asked the following questions. ‘During the past 7 d, how many days did you eat all vegetables (about one and one-half serving of fresh vegetables) at lunch?’ ‘During the past 7 d, how many days did you drink enough plain water daily?’ The responses ranged from ‘0 d’ (scoring 0) to ‘7 d’ (scoring 7). A higher score of vegetable eating means that students ate higher levels of vegetables, while a higher score of plain water drinking means that students had higher levels of plain water drinking. In addition, students were asked ‘During the past 7 d, how many times did you drink a can or bottle of sugar-sweetened beverages such as Coke, milk, tea, or Pepsi?’ The response options included ‘I did not drink SSB during the past 7 d’ (scoring 1), ‘1 to 3 times during the past 7 d’ (scoring 2), ‘4 to 6 times during the past 7 d’ (scoring 3), ‘1 time per d’ (scoring 4), ‘2 times per d’ (scoring 5), ‘3 times per d’ (scoring 6), and ‘4 or more times per d’ (scoring 7). A higher score for the SSB drinking means that students drank higher levels of SSB.

### Physical activity

Physical activity behaviours included students’ participation in outdoor physical activity, and moderate-to-vigorous physical activity was measured by adapting questions from prior studies^(25,26)^. Students were asked ‘During the past 7 d, on how many days did you take outdoor physical activities for a total of at least 120 min d?’ and ‘During the past 7 d, on how many days did you participate in at least 30 min of physical activity/exercises per d? (Add up all the time you spent in any kind of physical activity that increased your heart rate and made you sweat and breathe hard, such as running, bicycling, swimming, basketball, dancing ….)’ The responses ranged from ‘0 d’ (scoring 0) to ‘7 d’ (scoring 7). A higher score of outdoor physical activity (≥2 h/d) means that students had higher levels of outdoor physical activity, while a higher score of moderate-to-vigorous physical activity (≥30 min/d) means that students had higher levels of moderate-to-vigorous physical activity. In addition, students were asked ‘During the past 7 d, how often did you take outdoor physical activities at school recess?’ The responses included ‘never’ (scoring 1), ‘seldom (1–2 recess times)’ (scoring 2), ‘sometimes (3–4 recess times)’ (scoring 3) and ‘usually (5–6 recess times)’ (scoring 4) to ‘always (every recess time)’ (scoring 5). A higher score of outdoor physical activities at school recess means that students had higher levels of outdoor physical activities at school recess.

### Covariate

Covariate variables included school level (primary school, middle school or high school), gender (male or female) and age.

### Data analysis

SAS software was used to perform the statistical analysis. Percentages and means were calculated for all variables. Chi-square tests were conducted to analyse students’ perceptions of their school environment by school level. A series of ANOVA tests were performed to compare students’ dietary behaviours and physical activity by school level. Multiple regression was used to examine the relationships of perceived school SSB ban policies, school SSB refusal campaigns, school plain water campaigns and students’ drinking of SSB and their compliance with the drinking of plain water after controlling for school level, as well as for students’ gender and age. Multiple regression was also conducted to examine the relationships of students’ perceptions of school vegetable/fruit campaigns and their consumption of vegetables. In addition, multiple regression models were conducted to examine the relationships of perceived school outdoor physical activity campaigns and SH150 programmes and students’ outdoor physical activity at school recess, outdoor physical activity (≥2 h/d) and moderate-to-vigorous physical activity (≥30 min/d).

## Results

### Students’ perceptions of their school’s environments by school level

The students’ perceptions of their school’s environment with respect to healthy eating and exercise are listed by school level in Table 1. The rate of students who perceived that their schools had implemented policies banning SSB and refusing to drink SSB was significantly higher in primary school (60·6 and 53·8 %, respectively) than in middle school (40·3 and 34·2 %, respectively) and high school (23·9 and 27·5 %). The rates of students who perceived that their schools had implemented initiatives promoting the drinking of plain water and the consumption of vegetables and fruit (five servings of fruits and vegetables a day and eating all the vegetables during school lunch) also were significantly higher in primary school (87·5 and 85·9 %, respectively) than in middle school (79·3 and 74·5 %, respectively) and high school (70·6 and 66·5 %, respectively). In addition, the rates of students who reported their school’s implementation of outdoor physical activity (≥ 120 min/d) and SH150 programme (exercise ≥ 150 min/week in school) were also significantly higher in primary school (80·8 and 78·9 %, respectively) than in middle school (71·2 and 71·7 %, respectively) and high school (59·9 and 57·5 %, respectively) (Table 1).

**Table 1:** Students’ perceptions of school environments that promotes healthy eating and physical activity by school level

	Primary school		Middle school		High school		*χ* ^2^
	*n*	%	*n*	%	*n*	%	*P*
School sugar-free policy	1474	60·6	1294	40·3	676	23·9	<0·0001
Refuse to drink sugar-sweetened beverages	1308	53·8	1098	34·2	775	27·5	<0·0001
Plain water campaign	2128	87·5	2546	79·3	1996	70·6	<0·0001
Vegetables and fruit eating campaign	2089	85·9	2401	74·8	1881	66·5	<0·0001
Outdoor physical activity ≥ 120 min/d campaign	1965	80·8	2288	71·2	1695	59·9	<0·0001
SH150 programme School exercise ≥ 150 min/week	1920	78·9	2304	71·7	1626	57·5	<0·0001

Primary school *n* 2433, middle school *n* 3212, high school *n* 2829.

### Dietary behaviours of students by school level

Students’ dietary behaviours and physical activity by school level are listed in Table 2. Primary school students reported drinking fewer SSB (Mean = 2·54) than that reported by middle school students (Mean = 2·69) and by high school students (Mean = 2·88). In contrast, primary school students reported higher levels of drinking enough water daily (5·27 d/week) compared with that reported by middle school students (4·74 d/week) and by high school students (4·43 d/week). In similar manner, primary school students reported higher levels of eating all vegetables (1·5 servings) at lunch (5·08 d/week) than that reported by middle school students (4·29 d/week) and by high school students (4·39 d/week).

**Table 2:** Students’ dietary behaviours and physical activity by school level

Variable (score range)	Primary school		Middle school		High school		ANOVA test
	Mean	SD	Mean	SD	Mean	SD	*P*
SSB drinking frequency (1–7)	2·54	1·42	2·69	1·45	2·88	1·42	<0·0001
Plain water intake days a week (0–7)	5·27	2·09	4·74	2·31	4·43	2·50	<0·0001
Lunch vegetables consumed (1·5 servings) days a week (0–7)	5·08	2·20	4·29	2·43	4·39	2·48	<0·0001
Outdoor physical activity at school recess (1–5)	3·41	1·22	3·24	1·19	2·86	1·08	<0·0001
Outdoor physical activity (≥2 h/d) days a week (0–7)	3·88	2·33	3·07	2·26	3·06	2·22	<0·0001
Exercise (≥30 min/d) days a week (0–7)	4·93	2·09	4·01	2·18	3·68	2·23	<0·0001

Primary school *n* 2433, middle school *n* 3212, high school *n* 2829.

### Physical activity of students by school level

In addition, primary school students (Mean = 3·41) and middle school students (Mean = 3·24) reported higher levels of outdoor physical activity at school recess than that reported by high school students (Mean = 2·86). Primary school students also reported higher levels of outdoor physical activity (more than 120 min/day) (3·88 d/week) than that reported by middle school students (3·07 d/week) and by high school students (3·07 d/week). In addition, primary school students reported higher levels of moderate-to-vigorous physical activity (more than 30 min/d) (4·93 d/week) than that reported by middle school students (4·01 d/week) and by high school students (3·68 d/week).

### Relationships between school environment and students’ dietary behaviours

Multiple regression results showed that after controlling for school level and for students’ gender and age, students’ perceptions of school policies banning SSB and refusing the drinking of SSB were negatively associated with the drinking of SSB. Students’ perceptions of campaigns promoting the drinking of plain water and banning SSB were positively associated with sufficient drinking of plain water daily. In addition, students’ perceptions of campaigns promoting the consumption of five servings of vegetables and fruit and eating all vegetables at school lunch was positively associated with students’ vegetable intake. However, the multivariate analyses showed that compared with primary school students, middle and high school students were less likely to drink plain water and eat all vegetables at school lunchtime, while boys were more likely than girls to drink SSB and eat all vegetables at school lunchtime (Table 3).

**Table 3:** Relationships of students’ perceptions of school environments and dietary behaviours

	Drink SSB		Drink plain water		Eat vegetables at lunch	
	*β*	*P*	*β*	*P*	*β*	*P*
Intercept	2·55	<0·0001	3·46	<0·0001	3·15	<0·0001
Gender (boy = 1, girl = 0)	0·40	<0·0001	−0·03	0·2757	0·30	<0·0001
Age	0·01	0·6349	−0·29	0·0002	0·02	0·4932
School level						
(middle = 1, primary = 0)	0·05	0·3229	−0·29	0·0002	−0·63	<0·0001
(high = 1, primary = 0)	0·17	0·0547	−0·33	0·0174	−0·42	0·0034
School sugar-sweetened beverage ban policy (yes = 1, no = 0)	−0·09	0·0286	0·01	0·9053		
School sugar-sweetened beverage refusal campaign (yes = 1, no = 0)	−0·28	<0·0001	0·16	0·0072		
School plain water campaign (yes = 1, no = 0)	−0·11	0·0051	1·97	<0·0001		
School vegetable/fruit campaign (yes = 1, no = 0)					1·83	<0·0001

*n* 8245.

### Relationships between school environments and students’ physical activity

Multiple regression results showed that after controlling for school level and for students’ gender and age, students’ perceptions of their schools’ implementation of campaigns promoting outdoor physical activity at school recess (120 min/d) and SH150 programmes (moderate-to-vigorous physical activity 150 min/week at school) were positively associated with outdoor physical activity at school recess. Students’ perceptions of outdoor physical activity and exercise environments were also positively associated with daily outdoor physical activity. In addition, students’ perceptions of outdoor physical activity and exercise environments were positively associated with daily moderate-to-vigorous physical activity (Table 4).

**Table 4:** Relationships of students’ perceptions of school environments and physical activity behaviours

	Outdoor physical activity at school recess		Outdoor physical activity (≥2 h/d)		Moderate-to-vigorous physical activity (≥30 min/d)	
	*β*	*P*	*β*	*P*	*β*	*P*
Intercept	3·25	<0·0001	2·72	<0·0001	3·82	<0·0001
Gender (boy = 1, girl = 0)	0·26	<0·0001	0·53	<0·0001	0·64	<0·0001
Age	−0·04	0·0008	−0·03	0·1837	−0·05	0·0550
School level						
(middle = 1, primary = 0)	−0·03	0·5393	−0·61	<0·0001	−0·70	<0·0001
(high = 1, primary = 0)	−0·18	0·0201	−0·31	0·0354	−0·64	<0·0001
School outdoor physical activity campaign (yes = 1, no = 0)	0·44	<0·0001	0·97	<0·0001	0·90	<0·0001
SH150 programme (yes = 1, no = 0)	0·26	<0·0001	0·65	<0·0001	0·76	<0·0001

*n* 8245.

## Discussion

This study found that students’ perceptions of school policies that banned the drinking of SSB and encouraged the drinking of plain water were positively associated with students’ drinking of plain water and negatively associated with their consumption of sugary drinks. Studies conducted in the Netherlands, the USA and Mexico have indicated that school plain water drinking promotional campaigns/programmes also had positive effects on increasing children’s consumption of plain water and reducing the intake of SSB^(18)^ and were associated with a significant increase in the consumption of fruits and vegetables^(30)^. A European study found that accessibility to healthy foods in schools differs widely across countries, and those researchers suggested the implementation of uniform school policies to tackle unhealthy school nutrition environments throughout a given country^(31)^. These study results^(18,30,31)^ and the present study support that all primary, middle and high schools should implement sugar-free policies, request that teachers not offer SSB as a reward for students’ achievements and encourage students to drink plain water to reduce the consumption of SSB and increase the drinking of plain water.

In addition, this study also found that a student’s perception that their school had implemented a campaign promoting both the daily consumption of five fruits and vegetables and the need to eat all vegetables served at school lunches was positively associated with an increase in their consumption of vegetables. A New Zealand study also found that students’ perceptions of a school’s healthy nutrition climate had a positive influence on adolescents’ fruit and vegetable intake^(32)^. The US study indicated that students attending schools implementing healthy eating policies showed a significant increase in the consumption of fruits and vegetables and a decrease in the consumption of sugary drinks^(33)^, while a study indicated that student awareness of a school fruit and vegetable campaign/programme was associated with an increase in fruit and vegetable consumption^(23)^. In a global survey, countries with school policies promoting the consumption of fruit and vegetables reported an adequate consumption of fruit and vegetables among adolescents^(34)^. Studies have indicated that a combination of education, diet, physical activity and promoting a healthy food environment was most effective in preventing children from becoming overweight^(35,36)^. Taiwan schools could combine healthy food policies in schools with campaigns to eat all vegetables offered at school meals and programmes promoting food and school gardening and nutrition education to increase the consumption of vegetables and fruit by students.

This study found that students’ perceptions of school outdoor physical activity at school recess and school exercise environments were positively associated with students’ outdoor physical activity and daily moderate-to-vigorous physical activity. A review study found that school physical activity policies and promoting a physical and social-environmental climate were associated with adolescent physical activity^(22)^. Another study also showed that the school social environment was associated with adolescent physical activity^(37)^. Prior study has indicated that school physical activity policies (i.e. break time length), physical activity programmes and exercise environments significantly increased adolescent physical activity^(22)^. A Canadian study indicated that after implementation of the guidelines for Food and Beverage Sales and Daily Physical Activity, fewer schools provided SSB and schools were more likely to meet 150 min/week of physical education^(38)^. Finland also showed that school-initiated physical activity programmes were effective for promoting regular physical activity for large numbers of students^(39)^. Schools could implement ‘whole school’ approaches that include increases in school break times for physical activity, adding multiple physical activity programmes and promoting outdoor physical activity during school recess to enhance the physical activity and health equity of students.

Results of the present study showed that high school and middle school students had lower rates of healthy dietary behaviours and physical activity than primary school students, and that fewer high and middle school students recognised the enactment of school healthy food and exercise environments compared with primary school students. A longitudinal study indicated that transitioning from primary to secondary school has a negative impact on students’ physical activity^(40)^. The previously mentioned global survey also showed that, compared with adolescents aged 11–13 years, adolescents aged 16–17 years had higher odds of reporting more than three lifestyle risk factors^(8)^. That study recommended implementation of prevention strategies targeting clusters of lifestyle risk factors to help mitigate the burden of NCD^(8)^. Since dietary behaviours among children are the result of complex interactions between biological, social and environmental factors^(41)^, it is crucial to examine how family/community and schools could reasonably work together to foster a healthy eating and physical activity culture. Prior study has indicated that the Health Promoting Schools approach has had a positive effect on improving students’ dietary behaviour^(42)^, and studies have indicated that the strength of evidence is high for a combination of diet/physical activity interventions delivered in schools that are coordinated with both home and community^(36,43,44)^. In Taiwan, Health Promoting Schools programmes have lower levels of implementation in middle and high schools than in primary schools^(45,46)^, while these results were consistent with our findings that fewer middle and high school students reported healthy school policies and campaigns compared with primary school students. Governments could provide greater financial and technical support for schools to implement the Health Promoting Schools approach and increase parent and community involvement to sustain students’ healthy behaviours at school and home.

The present study had some limitations. First, about one-tenth of schools and students refused to participate in the survey. Hence, potential biases from the selection and refusal to participate must be considered. Second, this study used a cross-sectional design and causal inferences were precluded. Third, students’ health behaviours and perceptions of school environments were collected through self-reported questionnaires, which could lead to potential recall and social desirability bias. Fourth, although the Taiwan government enacted Food and Beverage Sales policies in school and implemented SH150 programmes, students might not be aware these policies, programmes or campaigns and might interpret the description in the questionnaire differently. Fifth, Taiwan school lunch programmes were not free but were inexpensive in most cities and counties, but few students chose not to join the school lunch programme. The amount of vegetables in students’ lunchboxes brought from home or bought from school cafeteria or outside might be different from the amounts the school lunch programmes provide. Sixth, the measurement of sufficient drinking of plain water daily was based on students’ perceptions. Since students might not know how much daily water drinking is enough, future study could ask how much water students drink. Finally, since school health policies and environments were different at different school levels and different counties/cities, the impact might be different. However, the present study results found that the relationships of students’ perceptions of healthy school policies/campaigns were significantly related to students’ healthy eating and physical activity at all school levels. Future study could further examine the effects of school policies and environments on students’ health behaviours by different school level and in different counties/cities. Despite these limitations, the strength of this study was a large nationally representative sample size that was used to examine the relationships of students’ perceptions of school policies that eliminate SSB, promote the drinking of plain water, encourage the consumption of vegetables and fruit, and establish physical activity environments.

## Conclusions

This study highlights the importance of students’ perceptions of healthy school policies and environments to enhance students’ healthy eating and physical activity. This study revealed that compared with middle school and high school students, more primary school students reported that schools have implemented policies that eliminate SSB, promote the drinking of plain water, encourage the consumption of five servings of vegetables and fruits a day including all vegetables served in school lunches, and implement outdoor physical activities and the SH150 programme. Primary school students reported less sugary drinking, more plain water drinking, increased vegetable/fruit intake and greater levels of physical activity than middle and high school students. The multiple regression results indicated that after controlling for school level, gender and age, students’ perceptions of a school sugar-free environment were negatively related to students’ SSB consumption. Students’ perceptions of a school’s campaigns to promote the drinking of plain water and refusing to allow the drinking of SSB were positively related to students’ daily plain water intake and negatively related to the consumption of SSB. Students’ perceptions of a school’s vegetables/fruit campaigns were positively related to students’ consumption of vegetables. In addition, students’ perceptions of a school’s outdoor physical activity environment and the SH150 programme were positively related to student participation in outdoor physical activities and exercise.
